# A 14-3-3 Protein Ca16R Acts Positively in Pepper Immunity against *Ralstonia solanacearum* by Interacting with CaASR1

**DOI:** 10.3390/plants13101289

**Published:** 2024-05-07

**Authors:** Sheng Yang, Meiyun Wan, Xingge Cheng, Qing Cheng, Huolin Shen

**Affiliations:** 1 Department of Vegetable Science, College of Horticulture, China Agricultural University, Beijing 100193, China; 2022019@cau.edu; 2 College of Agriculture, Fujian Agriculture and Forestry University, Fuzhou 350002, China; 1210102021@fafu.edu.cn (M.W.); 1210102001@fafu.edu.cn (X.C.)

**Keywords:** *R. solanacearum*, pepper, 14-3-3, ASR

## Abstract

Although 14-3-3 proteins have been implicated in plant growth, development, and stress response, their roles in pepper immunity against *R. solanacearum* remain poorly understood. In this study, a 14-3-3-encoding gene in pepper, *Ca16R*, was found to be upregulated by *R. solanacearum* inoculation (RSI), its silencing significantly reduced the resistance of pepper plants to RSI, and its overexpression significantly enhanced the resistance of *Nicotiana benthamiana* to RSI. Consistently, its transient overexpression in pepper leaves triggered HR cell death, indicating that it acts positively in pepper immunity against RSI, and it was further found to act positively in pepper immunity against RSI by promoting SA but repressing JA signaling. Ca16R was also found to interact with CaASR1, originally using pull-down combined with a spectrum assay, and then confirmed using bimolecular fluorescence complementation (BiFC) and a pull-down assay. Furthermore, we found that CaASR1 transient overexpression induced HR cell death and SA-dependent immunity while repressing JA signaling, although this induction and repression was blocked by *Ca16R* silencing. All these data indicate that Ca16R acts positively in pepper immunity against RSI by interacting with CaASR1, thereby promoting SA-mediated immunity while repressing JA signaling. These results provide new insight into mechanisms underlying pepper immunity against RSI.

## 1. Introduction

The coevolution with various pathogens has equipped plants with elaborate immune mechanisms to activate rapid and efficient defense responses against pathogen attacks. These defense responses are regulated by complicated signaling networks and are initiated upon perception of pathogen infection by plants with various immune receptors. Ca^2+^ signaling; hormones including SA (Salicylic acid), JA (jasmonic acid), and ET (Ethylene); ROS including H_2_O_2_; MAPK cascades; and transcriptional cascades have been found to be involved in and shared by immune signaling initiated by plants’ immune receptors upon perception of pathogens [1,2,3,4]. Moreover, defense responses of a different nature that are activated to protect plants from attacks of different pathogens are finely regulated by the signaling networks made up of these components, and protein–protein interaction is one of the main methods of plant immune signal transmission [5,6], with the targeting proteins being activated or repressed through the modification of phosphorylation, methylation, or acetylation [7]. However, the underlying mechanisms remain to be elucidated.

14-3-3 proteins are phosphoserine-binding proteins and constitute a family of conserved proteins present in all eukaryotic organisms. The members of this family act as adaptors for protein–protein interactions, regulators of the localization of proteins or their activities [8], and thus crucial regulators of a wide range of target proteins in signal transduction in plant growth, development, and defense response to stress. 14-3-3 proteins have been found to be involved in the regulation of plant immunity [9,10,11,12,13,14] by targeting NLRs [15], MAPKKKs [9,14,15], substrates of CDPKs [16], RIN4 and GCN4 [17], and transcription factors such as GRAS proteins (by regulating its stability) [18]. Their roles as crucial regulators in plant immunity is reflected in their frequent attack or hijacking by pathogen effectors to weaken plant immune responses or increase pathogen virulence [10,19,20,21,22,23,24]. However, the roles of 14-3-3 proteins in plant immunity and the underlying mechanisms are not fully understood.

Pepper (*Capsicum annuum*) belongs to the Solanaceae and is of great agricultural importance. Bacterial wilt caused by *Ralstonia solanacearum* is one of the most important soil-borne diseases in the production of pepper and other solanaceous crops, especially in tropical or subtropical regions, causing heavy loss in the productivity of these crops [25,26]. According to our previous studies, transcription factors including CaWRKY27b [27], CaWRKY28 [28], CaWRKY40 [29], CabZIP23 [30], CabZIP63 [30], CaNAC2c [31], and CaNAC029 [31], as well as other proteins such as CaCDPK29 [27] and chromatin remodeling-related CaSWC4 [32], are involved and constitute regulatory networks in pepper immunity against *R. solanacearum* and also participate in pepper thermotolerance. CaWRKY40 and CabZIP23 act crucially in this network, with CaWRKY40 being directly regulated by CabZIP23, to activate pepper immunity and thermotolerance that are distinct in their nature. Both CaWRKY40 and CabZIP63 are modulated by other regulatory proteins post-translationally, for example, to activate pepper immunity, CaWRKY40 is regulated by CaWRKY28 [28] and CabZIP63 is modulated by CaASR1, an abscisic acid-, stress-, and ripening-inducible protein [33]. In addition, it has recently been found that the 14-3-3 proteins SlTFT4 and SlTFT7 have been targeted by *R. solanacearum* type III effectors to suppress host immunity [19,20], implying that 14-3-3 proteins might be involved in pepper immunity against *R. solanacearum*. However, no 14-3-3 protein has been found to play a role in pepper immunity against *R. solanacearum.* In the present study, a gene whose encoding amino acid sequence showed the highest similarities to 16R, among all of the 14-3-3 proteins in potato, was found to act positively in pepper immunity against *R. solanacearum* by interacting with CaASR1.

## 2. Results

### 2.1. Sequence Analysis of Ca16R

**Figure 1:**
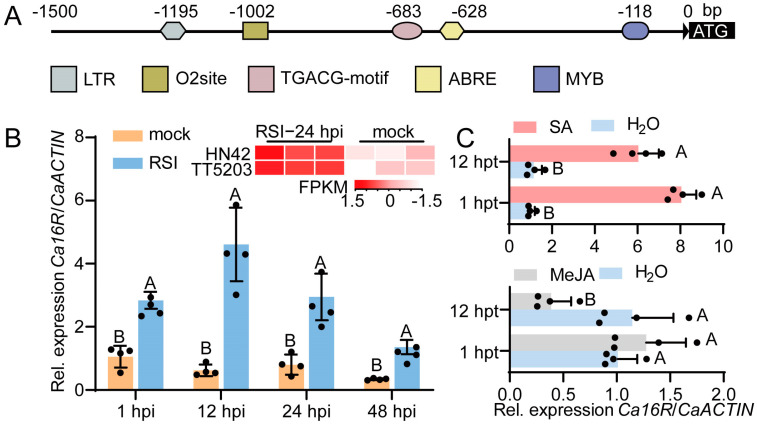
*Ca16R* was upregulated by *R. solanacearum* inoculation. (**A**) Schematic diagram of the distribution of cis-elements on the promoter of *Ca16R*. (**B**) *Ca16R* was upregulated by *R. solanacearum* inoculation from 1 to 48 hpi, reaching the highest expression level at 12 hpi; FPKM: fragments per kilobase of exon model per million mapped fragments. (**C**) *Ca16R* was upregulated by the exogenous application of SA but downregulated by exogenously applied MeJA. Data in (**B**,**C**) represent the mean ± SD from four independent experiments. Error bars indicate SD. Uppercase letters above the bars indicate significant differences (*p* < 0.01) calculated with Fisher’s protected *t*-test.

In a set of RNA-seq data in pepper plants challenged with *R. solanacearum* infection [34], a gene-encoding 14-3-3 protein was found to be upregulated by *R. solanacearum* infection (RSI) at 24 hpi (hours post inoculation) from two pepper inbred lines (Figure 1B). This implies its possible role in pepper immunity against *R. solanacearum*, since a gene that is transcriptionally regulated by a pathogen infection is generally involved in plant immunity against this pathogen [29]. The 14-3-3 protein (XP_016550580.1) exhibits high amino acid sequence similarity to 16R among all of the 14-3-3 proteins in potato and to its orthologues in other Solanaceae (Figure S1), so we named it Ca16R. To test whether *Ca16R* was upregulated by RSI, the enrichment of cis-elements related to plant immunity was scanned and it was found that a TGACG motif, which is bound by TGACG motif-binding (TGA) transcription factors involved in the regulation of the salicylic acid (SA) and methyl jasmonate (MeJA) signaling pathways [35], was present in the promoter of *Ca16R* (Figure 1A). Consistently, *Ca16R* was found to be upregulated by RSI from 1 hpi to 48 hpi (Figure 1B) and by exogenously applied SA but downregulated by the exogenous application of MeJA at 12 hpt (Figure 1C). In addition, the subcellular localization of Ca16R in epidermal cells of *Nicotiana benthamiana* leaves was assayed, and the results showed that Ca16R might locate in the cytoplasm and nuclei (Figure S2). All these data indicate that *Ca16R* is upregulated by RSI, probably in an SA-signaling-dependent manner.

### 2.2. Silencing of Ca16R by Means of Virus-Induced Gene Silencing Reduced Pepper Resistance to RSI

**Figure 2:**
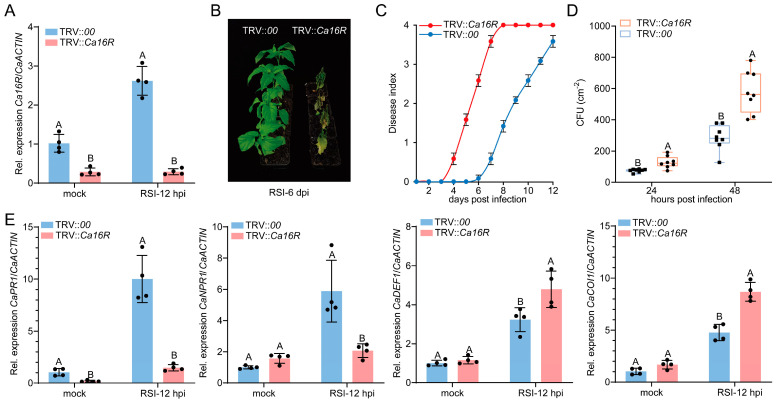
Silencing of *Ca16R* significantly reduced pepper resistance to *R. solanacearum* inoculation. (**A**) The transcript level of *Ca16R* in *R. solanacearum* TRV::*Ca16R* plants inoculated after 12 h is only 10% of that in the wild-type plants. (**B**) TRV::*Ca16R* pepper plants exhibited a significantly lower level of resistance to RSI compared to the wild-type plants. (**C**) TRV::*Ca16R* pepper plants exhibited a significantly higher level of dynamic disease index from 3 to 12 dpi compared to the wild-type plants. (**D**) TRV::*Ca16R* pepper plants provided a higher level of bacterial growth than the wild-type plants. Data are shown as the mean ± standard error of eight replicates. Different uppercase letters above the bars indicate significant differences (*p* < 0.01) calculated using Fisher’s protected *t*-test; CFU/cm^2^: the number of *R. solanacearum* colonies in 1 cm^2^ of leaf. (**E**) The *R. solanacearum* TRV::*Ca16R* pepper plants inoculated after 12 h exhibited a lower expression level of *CaPR1* and *CaNPR1* but a higher expression level of *CaDEF1* and *CaCOI1*. Data in (**A**,**E**) represent the mean ± SD from four independent experiments. Error bars indicate SD. Uppercase letters above the bars indicate significant differences (*p* < 0.01) calculated using Fisher’s protected *t*-test.

The upregulation of *Ca16R* by RSI and the exogenous application of SA indicate its involvement in pepper immunity against RSI. To test this possibility, the effect of *Ca16R* silencing on pepper resistance to RSI was assayed, the success of *Ca16R* in pepper plants by VIGS was confirmed, and the transcript level of *Ca16R* in RSI-challenged TRV::*Ca16R* plants at 12 hpi was only 10% of that in the TRV::*00* plants (Figure 2A). To detect the specificity of the *Ca16R* silencing, we tested the transcript levels of *Ca14-3-3 6* (Capana05g000289), *Ca14-3-3 2* (Capana12g001301), and *Ca14-3-3 C* (Capana04g000995), which belong to different 14-3-3 subfamilies in the pepper genome. We found that the silencing of *Ca16R* did not reduce the transcript levels of the tested genes compared to the mock treatment (Figure S3), indicating the specificity of *Ca16R* silencing. The VIGS results showed that TRV::*Ca16R* plants exhibited reduced resistance to RSI compared to the TRV::*00* plants, a higher dynamic disease index from 3 to 12 dpi was found in TRV::*Ca16R* plants than that in TRV::*00* plants (Figure 2B,C), and higher bacterial growth was found in RSI-challenged TRV::*Ca16R* plants at 24 and 48 hpi than the wild-type plants (Figure 2D). Consistent with the results of the expression of *Ca16R* upon exogenous application of SA and MeJA, we found that SA-signaling-dependent *CaPR1* and *CaNPR1* were repressed by *Ca16R* silencing, while the expression of JA-dependent *CaDEF1* and *CaCOI1* was upregulated by *Ca16R* silencing in pepper plants. These data indicate that Ca16R acts positively in pepper immunity against RSI in an SA-signaling-dependent manner and also plays a role in the antagonism between SA and JA signaling.

### 2.3. Transient Overexpression of Ca16R Enhanced HR (Hypersensitive Response) Cell Death and SA-Signaling-Dependent PR Genes While Downregulating JA-Dependent PR Genes in Pepper Plants

**Figure 3:**
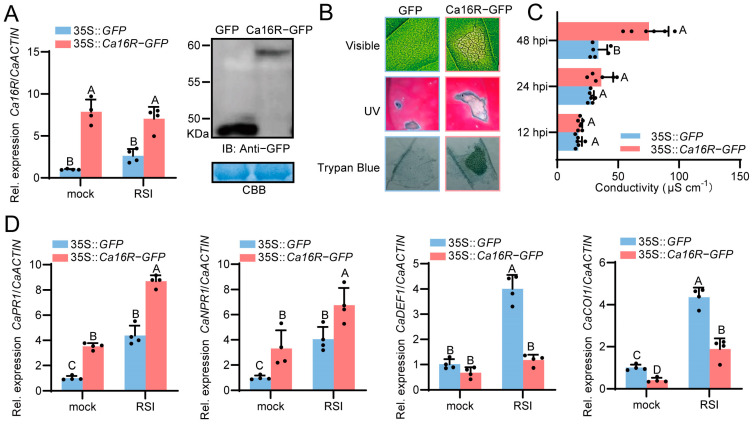
Transient overexpression of Ca16R triggered clear HR cell death and upregulated the expression of SA-dependent genes while repressing JA-dependent immunity-related genes. (**A**) Confirmation of the transient overexpression of Ca16R-GFP with RT-qPCR and with Western blotting using anti-GFP. (**B**) Transient overexpression of Ca16R after 96 hpi triggered HR cell death displayed by darker trypan blue staining. (**C**) Transient overexpression of Ca16R triggered higher ion leakage displayed by conductivity at 48 hpi. Data are shown as the mean ± standard error of six replicates. Different uppercase letters above the bars indicate significant differences (*p* < 0.01) based on Fisher’s protected *t*-test; µS/cm: microsiemens per centimeter. (**D**) SA-dependent *CaPR1* and *CaNPR1* were upregulated but JA-dependent *CaDEF1* and *CaCOI1* were downregulated by the transient overexpression of Ca16R. Data in (**A**,**D**) represent the mean ± SD from four independent experiments. Error bars indicate SD. Uppercase letters above the bars indicate significant differences (*p* < 0.01) calculated using Fisher’s protected LSD test. The RSI pepper leaves were harvested 12 h after *R. solanacearum* inoculation.

To confirm the results of the VIGS assay, we studied the effect of the transient overexpression of Ca16R in leaves of pepper plants using the agroinfiltration method [30]. The success of Ca16R-GFP transient overexpression in pepper leaves was assayed using RT-qPCR as well as Western blotting using anti-GFP (Figure 3A). HR cell death was found to be produced in pepper leaves by the transient overexpression of Ca16R; consistently, a darker trypan blue staining was also found to be produced by Ca16R transient overexpression (Figure 3B). Correspondingly, the higher ion leakage displayed by conductivity (Figure 3C) was found to be produced by the transient overexpression of Ca16R. In addition, the SA-signaling-dependent *CaPR1* and *CaNPR1* were upregulated, while the tested JA-dependent genes *CaDEF1* and *CaCOI1* were downregulated by the transient overexpression of Ca16R (Figure 3D). All these data confirmed the results of the VIGS that showed that Ca16R acts positively in pepper immunity against RSI.

### 2.4. Ectopic Overexpression of Ca16R Promoted Resistance of Nicotiana benthamiana to RSI

**Figure 4:**
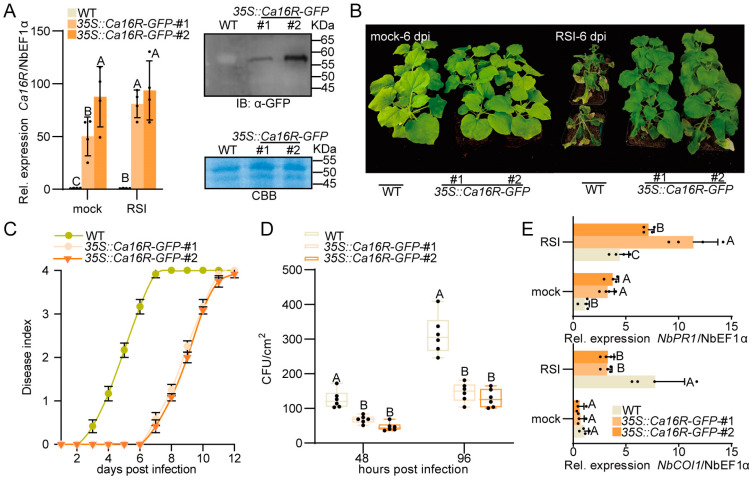
Overexpression of Ca16R-GFP enhanced the resistance of *N. benthamiana* to RSI. (**A**) The success of Ca16R with RT-qPCR and with Western blotting using anti-GFP. (**B**) The plants of Ca16R-overexpressing *N. benthamiana* lines #1 and #2 exhibited enhanced resistance to RSI compared to the wild-type plants. (**C**) The plants of Ca16R-overexpressing *N. benthamiana* lines #1 and #2 exhibited a lower disease index from 2 to 12 dpi compared to the wild-type plants. Data are shown as the mean ± standard error of twelve replicates. (**D**) The *R. solanacearum*-inoculated plants of Ca16R-overexpressing *N. benthamiana* lines #1 and #2 supported a lower level of bacterial growth at 48 and 96 hpi compared to the wild-type plants. Uppercase letters above the bars indicate significant differences between mean values (*p* < 0.01), as calculated with an LSD test. The center line represents the median value and the boundaries indicate the 25th percentile (upper) and the 75th percentile (lower). Whiskers extend to the largest and smallest values. (**E**) The *R. solanacearum*-inoculated plants of Ca16R-overexpressing *N. benthamiana* lines #1 and #2 exhibited a higher level of *NbPR1* expression and a lower level of *NbCOI1* expression compared to the wild-type plants. Data in (**A**,**E**) represent the mean ± SD from four independent experiments. Error bars indicate SD. Uppercase letters above the bars indicate significant differences (*p* < 0.01) calculated using Fisher’s protected LSD test. The RSI *N. benthamiana* leaves were harvested 12 h after *R. solanacearum* inoculation.

To further confirm the results that indicate that Ca16R acts positively in pepper immunity against RSI and to determine whether the function of Ca16R is conserved in Solanaceae, we generated Ca16R-overexpressing *N. benthamiana* T3 homozygous lines, in which two lines (#1 and #2) were randomly selected for further assay. The success of the ectopic overexpression of Ca16R was confirmed with RT-qPCR as well as Western blotting using anti-GFP (Figure 4A); the two overexpressing lines exhibited increased resistance to RSI compared to the wild-type plants (Figure 4B) and a lower level of dynamic disease index from 2 to 12 dpi (Figure 4C). In addition, a lower level of bacterial growth was found in the RSI-challenged Ca16R-overexpressing *N. benthamiana* plants than that in the wild-type plants at 48 and 96 hpi (Figure 4D), and a higher level of *NbPR1* but a lower level of *NbCOI1* was found in the RSI-challenged Ca16R-overexpressing *N. benthamiana* plants than that in the wild-type plants (Figure 4E). All these data indicate that Ca16R acts in pepper immunity against RSI, and this mechanism might be conserved in Solanaceae.

### 2.5. Ca16R Interacted with CaASR1

**Figure 5:**
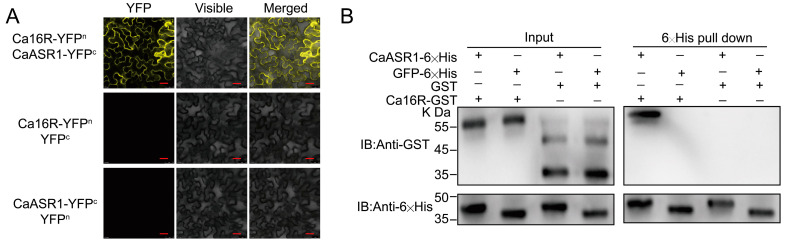
Ca16R interacted with CaASR1 using BiFC and a pull-down assay. (**A**) The data from BiFC showed that CaASR1 interacted with Ca16R in plasma membrane and cytoplasm in epidermal cells of *N. benthamiana* leaves; bar is 25 um. (**B**) The data from the pull-down assay showed that CaASR1 interacted with Ca16R. Ni Smart beads and CaASR1-6×His were incubated with Ca16R-GST for three hours at 4 °C with gentle rotation. Eluting the bound proteins from the beads, they were found using either an anti-GST or an anti-6×His antibody.

As 14-3-3 proteins fulfill their function mainly by interacting with other proteins, to isolate the possible interactors of Ca16R, we performed a coIP (co-immunoprecipitation) combined with a spectrum mass assay, and a subset of putative interacting proteins of Ca16R were isolated and determined (Table S2). CaASR1, which was previously found to act positively in pepper immunity against RSI by interacting with CabZIP63, was found. Its interaction with Ca16R was first confirmed with BiFC (Figure 5A), the results of which showed that the CaASR1-Ca16R interaction occurred outside the nuclei. The interaction between CaASR1 and Ca16R was further confirmed by means of a pull-down assay using prokaryotically expressed CaASR1-6×His and Ca16R-GST (Glutathione S-transferases) (Figure 5B).

### 2.6. CaASR1 Was Promoted to Activate SA-Signaling-Mediated Immunity with Ca16R

**Figure 6:**
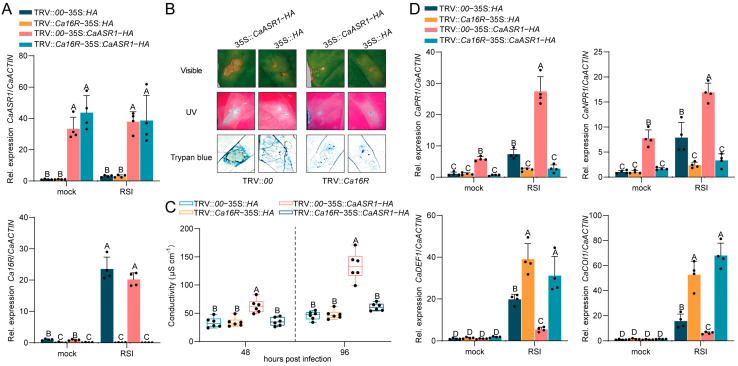
Transient overexpression of CaASR1 induced immunity, including HR cell death, but this induction was repressed by *Ca16R* silencing. (**A**) Using the RT-qPCR assay, the success of *CaASR1* transient overexpression and *Ca16R* silencing through virus-induced gene silencing in pepper plants. (**B**,**C**) Hypersensitive response-like cell death was observed by means of UV, trypan blue staining, and intensive ion leakage displayed by conductivity. CaASR1 was transiently overexpressed in WT and *Ca16R*-silenced pepper leaves, and silenced *Ca16R* weakened the hypersensitive response. Data are shown as the mean ± standard error of six replicates. Different uppercase letters above the bars indicate significant differences (*p* < 0.01) according to Fisher’s protected LSD test. (**D**) The activation of SA-dependent *CaPR1* and *CaNPR1* by CaASR1 was weakened by *Ca16R* silencing upon RSI at 48 hpi with RT-qPCR. In (**A**,**D**), the mean ± SD of four duplicate results are shown. Based on Fisher’s least significant difference (LSD) test, discrete capital letters on the bar graphs denote statistically significant differences (*p* < 0.01) between mean values. The RSI pepper leaves were harvested 12 h after *R. solanacearum* inoculation.

To test the possible role of the CaASR1-Ca16R interaction in pepper immunity against RSI, we studied the effect of *Ca16R* silencing on the immunity triggered by *CaASR1* transient overexpression, which was found in our previous study to activate SA-signaling-mediated immunity through physical interaction with CabZIP63 [30,33,36] in RSI-challenged pepper plants; the success of *CaASR1* transient overexpression and *Ca16R* silencing were confirmed using RT-qPCR (Figure 6A). The results showed that the transient overexpression of CaASR1 produced clear HR cell death, indicated by darker trypan blue staining (Figure 6B); consistently, ion leakage reflected by conductivity was found to be triggered by *CaASR1* transient overexpression (Figure 6C), but these were all significantly repressed by *Ca16R* silencing in pepper leaves. In addition, SA-dependent *CaPR1* and *CaNPR1* were upregulated, while JA-dependent genes including *CaDEF1* and *CaCOI1* were downregulated by the transient overexpression of *CaASR1* in TRV::*00* pepper leaves; the upregulation of *CaPR1* and the downregulation of *CaDEF1* and *CaCOI1* by the transient overexpression of *CaASR1* were also blocked by *Ca16R* silencing (Figure 6D). These results indicate that *CaASR1* and *Ca16R* synergistically activate SA-dependent immunity but repress JA-dependent immunity against RSI in pepper plants.

## 3. Discussion

Although 14-3-3 proteins have been implicated in plant immunity, their roles in pepper immunity against RSI remain poorly understood. We provided evidence that Ca16R, a 14-3-3 protein in pepper, has an active role in the defense of pepper against RSI by interacting with CaASR1.

### 3.1. Ca16R Acts Positively in Pepper Immunity against RSI

Our data showed that *Ca16R* silencing reduced pepper resistance to RSI (Figure 2). By contrast, the Ca16R transient overexpression triggered clear HR cell death (Figure 3), and the ectopic overexpression of Ca16R consistently promoted the resistance of *N. benthamiana* to RSI (Figure 4). In addition, *Ca16R* was upregulated by RSI from 1 to 48 hpi and reached the maximum expression level at 12 hpi. Based on these data, we can speculate that in non-stressed pepper plants, due to the silencing of *Ca16R*, no immunity is activated; however, when the plants are challenged by RSI, the upregulated Ca16R activates SA immunity. These results are consistent with those of a previous study which found that 14-3-3s including SlTFT4 and SlTFT7 in tomato are targeted by effectors from *R. solanacearum* [20], indicating that multiple 14-3-3s might be involved in plant immunity against *R. solanacearum*. The immune response of plants is produced by a large amount of transcription reprogramming [37]. After plants perceive the invasion of pathogenic bacteria, a large number of positive regulatory factors of immune response will be activated by transcription, thus making plant cells enter a stress-resistant state [38]. These results also support the notion that a gene upregulated by a given pathogen might play a role in plant immunity against this pathogen [29,39,40].

### 3.2. Ca16R Acts Positively in SA-Dependent but Negatively in JA-Dependent Pepper Immunity during Its Response to RSI at an Early Stage

As a hemibiotrophic pathogen, *R. solanacearum* infects the host plants biotrophically and transforms into a necrotrophic pathogen when the tissue of the host plants are destroyed [41]. Thus, plants might employ SA-signaling-mediated immunity to protect themselves from *R. solanacearum* attack while repressing JA-mediated immunity [34] due to its antagonistic effect on SA signaling. SA signaling has mainly been found to be involved in plant immunity against pathogens of a biotrophic lifestyle, while JA-signaling-mediated immunity is mainly employed by plants to protect themselves from attack from necrotrophic pathogens [42], and there is antagonism in general between SA- and JA-mediated signaling [43,44,45]. Our data also showed that Ca16R act positively in pepper immunity against RSI by activating SA-dependent *CaPR1* and *CaNPR1* [46,47] while repressing JA-responsive *CaDEF1* [48] and *CaCOI1* [49,50], which is in agreement with the data that indicate that clear HR cell death was triggered by Ca16R transient overexpression and is also consistent with the upregulation of Ca16R during the early stage of RSI from 1 to 48 hpi, with its maximum expression level found at 12 hpi. Thus, it can be speculated that Ca16R acts positively in SA-dependent but negatively in JA-dependent pepper immunity during its response to RSI at the early stage. The antagonism between these defense hormones shapes the signal transduction pathway in plant immune response [44]. This is related to plant response to different lifestyle pathogens. Cell death induced by SA accumulation can effectively prevent pathogens from plundering and spreading nutrients to hosts when dealing with biotrophic and hemibiotrophic pathogens, while the situation is completely opposite when dealing with necrotrophic pathogens [51]. It is important for plants to maintain the survival of cells and tissues [52]. The transient overexpression of Ca16R in pepper leaves accelerates cell death and supports the idea that it may be a member of SA signaling (Figure 3B). Similar to Ca16R, some members of SA signaling also maintain a correct immune response by inhibiting JA [53]. In conclusion, our findings suggest that Ca16R not only acts as a positive immune regulator, but also contributes to the accurate activation of pepper resistance to *R.solanacearum* infection.

### 3.3. Ca16R Potentiates SA Signaling but Represses JA Signaling through Physical Interaction with CaASR1

It has been found that 14-3-3 proteins fulfill their functions in regulation of plant immunity by interacting with client proteins as adaptor molecules stimulating protein–protein interactions, or as regulators to regulate the subcellular localization of proteins or their activity [14]. Our data showed that Ca16R interacts with CaASR1 (Figure 5), and CaASR1 was previously found to promote SA-signaling-mediated immunity by interacting with CabZIP63, thereby promoting the binding of CabZIP63 to its SA-dependent immunity-related target genes and their corresponding transcriptional activation [33]. Thus, it can be speculated that Ca16R activates SA-signaling-mediated immunity by interacting with CaASR1, which in turn causes CabZIP63 to activate SA-signaling-mediated immunity-related target genes. The repression of JA signaling might be due to the antagonistic effect of SA signaling [43,44,45].

Because CaASR1 interacts with CabZIP63 in the nucleus and with Ca16R outside the nucleus (Figure 5A), Ca16R, CaASR1, and CabZIP63 do not play a role in forming the same protein complex. We also found that Ca16R influences the expression of CabZIP63-targeted downstream genes (*CaPR1*, *CaNPR1*, and *CaDEF1*), and Ca16R also supports the function of CaASR1 in promoting SA and antagonizing JA. Therefore, we speculate that Ca16R affects the transcriptional regulation of CabZIP63 by affecting the function of CaASR1, which mediates the antagonism between SA and JA. Signal cascade mediated by phosphorylation modification is an important transmission mechanism of plant immune signals in the cytoplasm [54]. It works mainly through protein kinase, in which 14-3-3 protein plays an auxiliary role [55,56]. Ca16R may support the function of CaASR1 by affecting phosphorylation modification so that it can affect the transcriptional regulation of CabZIP63 in the nucleus. Given that CaCDPK15 [57] and CaCDPK29 [27] are involved in pepper immunity against RSI, and CDPKs and MAPKs coordinate with 14-3-3s in regulating diverse aspects of plant biology including metabolism, development, and stress responses [14,16], we speculate that some unidentified kinases might be involved in the functional relationship between Ca16R and CaASR1; however, further study is required to confirm this speculation.

Based on all the results, it can be concluded that Ca16R acts positively in pepper immunity by interacting with CaASR1 to activate SA signaling at the early stage during *R. solanacearum* infection.

## 4. Materials and Methods

### 4.1. Plant Materials and Pathogen Preparation

The pepper inbred lines HN42 and *Nicotiana benthamiana* were used in the present study; the seeds were sown and plants were cultivated in pots in a growth room or growth chamber under the same conditions described in our previous study [58]. *Ralstonia solanacearum* strain FJC100301 [29] was used and the activation and culture methods of *Ralstonia solanacearum* followed our previous study [58].

### 4.2. Inoculation of Pepper or Nicotiana benthamiana with R. solanacearum Cells

For the assay of the tolerance of plants to *R. solanacearum* inoculation, each pepper (6- to 8-leaf stage) or *N. benthamiana* (10- to 12-leaf stage) plant, with its roots being mechanically damaged, was inoculated using 0.5 mL of cell suspension of *R. solanacearum* (OD_600_ = 1.0) with root irrigation. The inoculated plants were placed in the growth room under the conditions of 28 °C, 90% humidity. For leaf inoculation with *R. solanacearum*, we inoculated leaves with 100 μL of *R. solanacearum* cell suspension (OD_600_ = 0.3) at each inoculating site using a syringe without needle.

### 4.3. Sequence Analysis and Primer Design

The ORF sequence of a given gene was found on the Solanaceae Genome Database (https://solgenomics.net/, accessed on 4 June 2023), and ORF and virus-induced gene-silencing (VIGS) primers were designed on DNAMAN6 software based on Gateway technology. In order to carry out amino acid homologous sequence alignment, we used the Blast function of NCBI (https://blast.ncbi.nlm.nih.gov/Blast.cgi) (accessed on 4 June 2023) to obtain its homologous sequence in other species. We selected and downloaded some homologous amino acid sequences from some Solanaceae plants and other non-Solanaceae plants, and used DNAMAN software for homologous sequence alignment and evolutionary tree analysis. Primers for RT-qPCR were designed using the NCBI Primer Blast function.

### 4.4. SA and MeJA Application

External spraying methods were used to treat SA and MeJA. The methods of external spraying SA (5 mM) and MeJA (100 mM) were performed following our previous study [34]. SA and MeJA solution as well as distilled water (ddH_2_O) as a negative control were evenly sprayed on the surface of pepper leaves using a sprayer, and transparent plastic bags were used for isolation. The leaves were harvested at the corresponding time point and used for subsequent experiments.

### 4.5. RNA Extraction and RT-qPCR Assay

The total RNA was isolated following the method in our previous study [27], using 2 mL RNase-free microcentrifuge tubes and three stainless beads and Tissue Lyser II (Qiagen, Dusseldorf, Germany) to disrupt plant material frozen by liquid nitrogen, using Trizol (Invitrogen, Carlsbad, CA, USA) and chloroform to isolate total RNA, and using isopropyl alcohol to precipitate RNA. The RNA was cleaned with 75% ethanol and then dissolved using ddH_2_O. The concentration and quality of RNA were determined with the NanoDrop 2000 (ThermoScientific, Massachusetts, MA, USA). Then, 50 ng RNA with DNA being digested, 250 ng of oligo dT(15) primer, and 200 unit reverse transcriptase and a One Step PrimeScript™ cDNA Synthesis Kit (TaKaRa, Shigo, Japan) were used for a reverse transcription reaction according to the following procedure: 42 °C, 60 min; 85 °C, 5 s; 4 °C to produce cDNA. To detect the relative transcript levels of the target genes, a Bio-Rad Real-Time PCR system (Bio-Rad Laboratories, Hercules, CA, USA) and SYBR Premix Ex Taq (Perfect Real Time; TaKaRa, Shigo, Japan) were used with the specific primer pairs listed in Table S1, using *CaActin* and *NbEF1a* as an internal reference gene to normalize the transcript expression levels [59]. The Livak method was used to analyze the data [60].

### 4.6. Subcellular Localization and Bimolecular Fluorescence Complementation (BiFC) Assay

The subcellular localization of Ca16R was performed following the method previously used [58]; GV3101 cells containing *35S:Ca16R-YFP* were grown, collected, and re-suspended with infection buffer; and an appropriate amount of the GV3101 cells was infiltrated into the leaves of the *Nicotiana benthamiana* plants. The GV3101 cells containing *35S:NbH2B-RFP* were co-infiltrated into leaves as a nuclear marker. To assay the interaction between Ca16R and CaASR1, GV3101 cells containing *pSPYCE-Ca16R* construct were mixed with *pSPYNE-CaASR1* construct at a 1:1 ratio, and then were infiltrated into *Nicotiana benthamiana* leaves. A laser scanning confocal microscope (TCS SP8; Leica Microsystems, Wetzlar, Germany) was used to capture images in subcellular localization and BiFC at 48 hpi. The emission filter and excitation wavelength were set to 488 nm (GFP), 510 nm (YFP), and 532 nm (RFP), with a band-pass of 500 to 550 nm (GFP and YFP) and 590 to 640 nm (RFP). The picture size was 150 × 150 μm, the format was 1024 × 1024, the objective lens was 100×, and the scanning frequency was 20 Hz for fluorescence photography and 144 Hz for cell observation.

### 4.7. Vector Construction

A specified region in a gene’s CDS or 3′ or 5′ UTR was amplified with PCR using specific primer pairs (Table S1) to create a vector for gene silencing; similarly, a full-length ORF of a Ca16R was amplified using PCR employing appropriate primer pairs (Table S1) to create a vector for overexpression. The PCR product was then cloned using a Gateway cloning system (Invitrogen, 11789020) into the entry vector pDONR207 with BP reaction. To construct a vector for overexpression, the subcellular location assay, and the BiFC assay, the ORF was further cloned from the entry vector to the pEarleyGate plasmid vectors pEarlyGate103, pEarlyGate201 [61]; pSPYCE, pSPYNE [62]; pDEST-17 (Invitrogen, 11803012) or pDEST-15 (Invitrogen, 11802014) using LR reaction. To construct a vector for the gene-silencing assay, the specific gene fragment in CDS or 3′ or 5′UTR of a given gene was cloned into the destination vector pPYL279 using LR reaction.

### 4.8. Virus-Induced Gene-Silencing (VIGS) Assay

To evaluate the role of Ca16R, VIGS was utilized following the protocol outlined in a previous study [27]. The conversion of GV3101 cells was accomplished through a process involving cold melting [63]. After mixing GV3101 cells carrying pTRV1 with GV3101 cells containing pTRV2:*00*, pTRV2:*CaPDS*, and pTRV2:*Ca16R* in a 1:1 proportion, the mixture was incubated at 28 °C with a speed of 60 rpm for 3 h. Subsequently, the combined bacterial solution was injected into the cotyledons of pepper seedlings that were 2 weeks old, and the seedlings were kept in conditions of darkness at 16 °C for 56 h.

### 4.9. Colony-Forming Units (CFU) and Disease Index Determination

To evaluate the growth of *R. solanacaerum* in plants, bacterial colony-forming units were measured in the plant material inoculated with *R. solanacearum*, and four biological replicates were performed in each group following the method described previously [58]. To measure the dynamic disease index in *R. solanacearum*-inoculated plants, the wilt symptoms of 12 plants were monitored from 1 to 12 hpi using a 0 to 4 score according to the method described previously [58].

### 4.10. Electrolyte Leakage and Trypan Blue

Electrolyte leakage was measured as previously described [33]. The harvested pepper leaves were washed with water, and then a hole punch was used to make leaf disks. The disks (6 mm in diameter) were excised from the leaves of pepper plants and incubated in 5 mL of ddH_2_O for 1 h at room temperature. The conductivity was measured using Mettler Toledo 326 (Mettler Toledo, Zurich, Switzerland).

Trypan blue staining was employed to assess HR cell death and was performed following the method used in our previous study [31].

### 4.11. Genetic Transformation of Nicotiana benthamiana

The genetic transformation of *Nicotiana benthamiana* followed the method used by Regner et al. [64] and Bardonn et al. [65]. Leaf discs were transformed with GV3101 cells containing objective vector and the acquired T_0_ plants were selected with 10% PPT (glufosinate, Sigma-Aldrich, Shanghai, China, 45520) and later confirmed by using PCR with specific primers (Table S1). The confirmed T_0_ plants were self-pollinated to produce the seeds of T1 lines, which were separately harvested; the acquired seeds were selected with 10% PPT during germination. Similarly, the seeds of T_2_ and T_3_ lines were acquired, and the homozygous T3 line plants were used for functional assays of the tested genes.

### 4.12. LC-MS/MS Analysis

To isolate interacting proteins of Ca16R, a LC-MS/MS approach was employed following the method used by us previously [27,58]. The *Agrobacterium tumefaciens* GV3101 cells containing *Ca16R-GFP* were transiently overexpressed in pepper leaves using agroinfiltration (the GV3101 cells containing *35S:GFP* as control); the agroinfiltrated leaves were harvested at 48 hpi for total protein isolation, Ca16R was IPed using anti-GFP, and the leaves were isolated and processed following Yang et al.’s method [58]. The samples were then analyzed using an LTQ-Orbitrap XL mass spectrometer (Thermo Fisher Scientific, Massachusetts, USA), as described earlier [66]. After dissolving the peptides in 10 μL of a 10% formic acid solution, an online sodium spray ion source was used for LC-MS/MS analysis. Proteome Discoverer 2.4 was used to import the original mass spectrometry collection files and retrieve them. (MS1 tolerance: 10 ppm; MS2 tolerance: 0.05 Da; missed cleavage: 2). The pepper Zunla-1 database (https://solgenomics.net/ftp/genomes/Capsicum_annuum/C.annuum_zunla/) (accessed on 12 April 2022) was searched against the peptide fragments, and a BLAST search of the UniProt database was used to annotate the protein functions.

### 4.13. Prokaryotic Expression

To obtain sufficient amounts of Ca16R-GST, CaASR1-6×His, pDEST-15, or pDEST-17, a plasmid harboring an appropriate vector was transformed into the *E. coli* strain BL21 (DE3). The bacterial transformation, bacterial cultivation, induction of fused protein, and protein isolation and purification were all performed following the method used by us previously [27]. The soluble fusion protein’s presence in the E. coli cell lysate supernatant was confirmed through the SDS-PAGE assay. Coomassie brilliant blue R250 (Sigma-Aldrich, Shanghai, China, 1.12553) was used to stain the electrophoretic SDS-PAGE gel, followed by de-colorization with a solution of 10% acetic acid and 5% ethanol to eliminate the background. Observing the gel bands determined the successful expression of the protein.

### 4.14. Pull-Down Assay

Taking the interaction between two proteins, for example, Ca16R and CaASR1, the prokaryotically expressed Ca16R-GST was incubated with CaASR1-6×His, using 6×His and GST as negative controls. The protein mixture was subjected to 50 μL BeaverBeads^TM^ IDA-Nickel (Beaver Biosciences Inc, Suzhou, China) magnetic beads to isolate and purify CaASR1-6×His. The purified protein mixture was added to a 5×SDS-PAGE loading buffer of 10 μL and denatured at 99 °C for 10 min. The protein mixture was separated using SDS-PAGE, and the presence of Ca16R was detected with Western blotting using anti-GST following the method we used previously [27].

### 4.15. Immunoblot Analysis

The pull-down eluted protein was examined with immunoblotting using anti-6×His and anti-GST (Abmart, Shanghai, China). The protein mixture was separated using SDS-PAGE and the protein in gel was transferred to a PVDF membrane activated by methanol, and the PVDF membrane containing the protein was sealed with TBST-5% non-fat milk powder. After that, the PVDF membrane was fully incubated with the antibody, and the excess antibody was cleaned using TBST. The PVDF membrane was subjected to chemiluminescence reaction with ECL luminescent (Biosharp, Guangzhou, China) substrate for 15 s, and the membrane was photographed under a GE ImageQuant LAS 4000 (General Electric, Chicago, IL, USA).

## Data Availability

The data that support the findings of this study are available in the Supplementary Materials.

## Supplementary Materials

The following supporting information can be downloaded at https://www.mdpi.com/article/10.3390/plants13101289/s1. Figure S1: Sequence comparison of Ca16R with its orthologues in other plant species; Figure S2: Subcellular localization of Ca16R in epidermal cells of *Nicotiana benthamiana* leaves; Figure S3: Specificity of *Ca16R* silencing; Table S1: Primers used in the present study; Table S2: List of the potential interacting partners of Ca16R revealed using IP-MS analysis.
