# Supplementary material for: A 14-3-3 Protein Ca16R Acts Positively in Pepper Immunity against Ralstonia solanacearum by Interacting with CaASR1
Source: Plants (Basel). 2024 May 7;13(10):1289. doi: 10.3390/plants13101289 (PMC11125135; doi:10.3390/plants13101289)
Supplement: Supplementary file 1 [file plants-13-01289-s001.zip › supplementary information.pdf]

Table S1. Primers used in this study.

	Gene	Forward primer	Reverse primer
Primers used for *Ca16R* study	***Ca16R***[1]	GGGGACAAGTTTGTACAAAAAAGCAGGCTT<br>C ATGGCGTCGCCACGTGAGGA	GGGGACCACTTTGTACAAGAAAGCTGGGTCTC<br>ATTCATTATCTGGTTTCG
	***Ca16R*-GFP**[2]	GGGGACAAGTTTGTACAAAAAAGCAGGCTT<br>C ATGGCGTCGCCACGTGAGGA	GGGGACCACTTTGTACAAGAAAGCTGGGTCTTC<br>ATTATCTGGTTTCG
	***Ca16R*-VIGS**[3]	GGGGACAAGTTTGTACAAAAAAGCAGGCTT<br>CCGCTGAAAGGAAAGAAGCTG	GGGGACCACTTTGTACAAGAAAGCTGGGTCTC<br>ATCCTGCATATCGGAGGT
Primers used for qPCR analysis study	***Ca16R*-qPCR**	CGTGTACATGGCAAAGCTGG	CCACGGTTAACTCCTCTCCG
	***CaASR1*-qPCR**	ACATGTCGGAGAACTCGGTG	TATCTTGTGCCTGTGTGCGT
	***CaPR1*-qPCR**	GCCGTGAAGATGTGGGTCAATGA	TGAGTTACGCCAGACTACCTGAGTA
	***CaNPR1*-qPCR**	ACTTCTTCGCCGACGCCAAG	GCCAACACATTCACCAGAGCATC
	***CaDEF1*-qPCR**	GTGAGGAAGAAGTTTGAAAGAAAGTAC	TGCACAGCACTATCATTGCATACAATTC
	***CaCOI1*-qPCR**	ATAATGAGCAAGCAGGAAA	TGTCAAGAAGGGCATAAAG
	***Ca14-3-3 6*-qPCR**	GACGAGCATCATGGCGGATA	ATCACCAGTAGCAGCCGAAC
	***Ca14-3-3 2*-qPCR**	GCCTACAAAGCTGCTCAGGA	CACGGTCGGGAGAGTTCAAA
	***Ca14-3-3 C*-qPCR**	GCGTGAGGAGAACGTGTACA	ATACGCCACGATGCTCTACG
	***CaACTIN*-qPCR**	AGGGATGGGTCAAAAGGATGC	GAGACAACACCGCCTGAATAGC
	***NbPR1*-qPCR**	ATGGGATTTGTTCTCTTTTCACA	TTAGTATGGACTTTCGCCTCT
	***NbCOI1*-qPCR**	CCCANAAGCATCCATCTCAC	GAAGATCTTGAATTGATGGC
	***NbEF-1a*-qPCR**	TGCTGCTGTAACAAGATGGATGC	GAGATGGGGACAAAGGGGATT

[1]Primers used for *Ca16R* full-length cloning

[2]Primers used for *35S:Ca16R-GFP* construct

[3]Primers used for pTRV:*Ca16R* construct

Supplemental figures

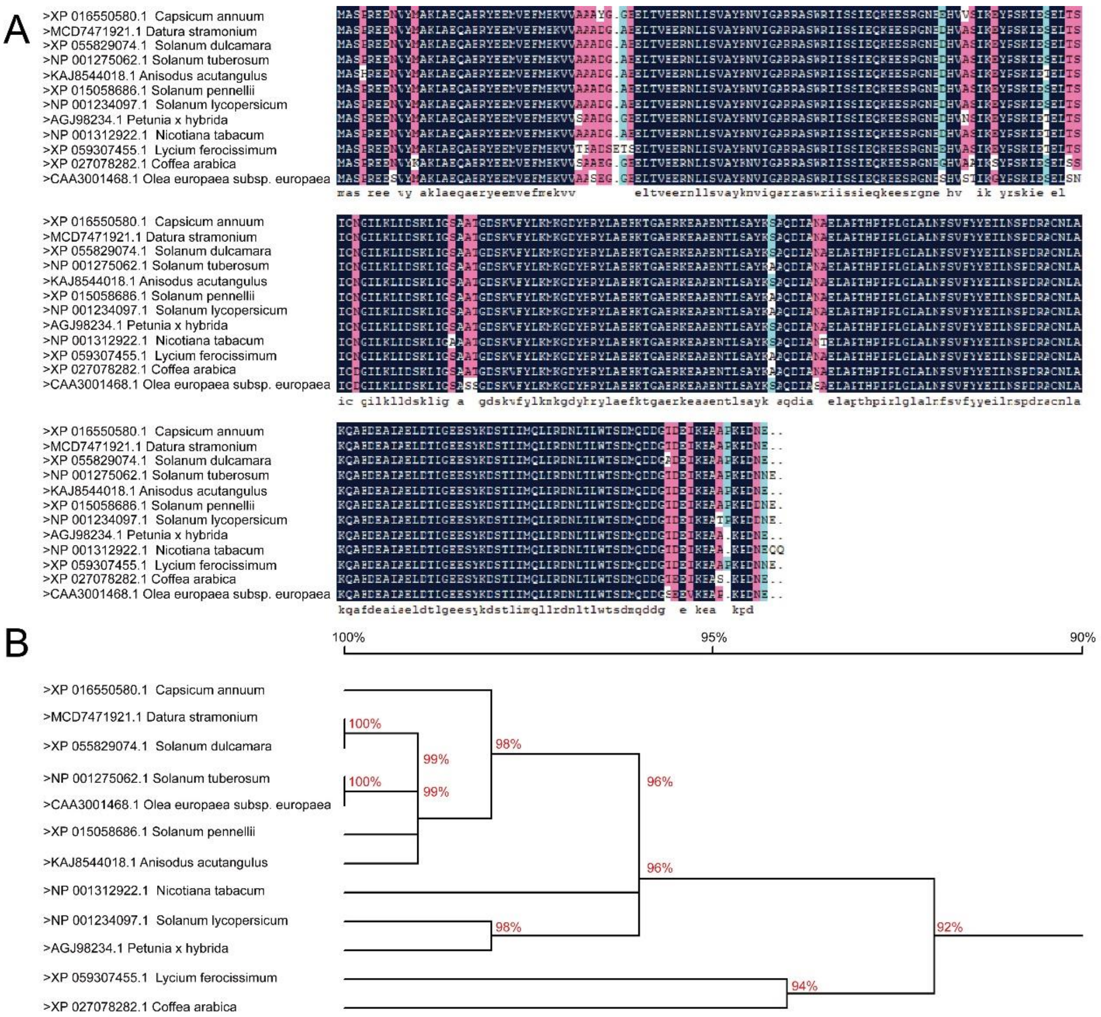

Figure S1. The sequence comparsion of Ca16R with its orthologues in other plant species. (**A**) Multiple alignment of deduced amino acid sequence of Ca16R with its orthologues in other plant species including *Datura stramonium* (MCD7471921.1); *Solanum dulcamara* (XP_055829074.1); *Solanum tuberosum* (NP_001275062.1); *Olea europaea* (CAA3001468.1); *Solanum pennellii* (XP_015058686.1); *Anisodus acutangulus* (KAJ8544018.1); *Nicotiana tabacum* (NP_001312922.1); *Solanum lycopersicum* (NP_001234097.1); *Petunia x hybrid* (AGJ98234.1); *Lycium ferocissimum* (XP_059307455.1); *Coffea Arabica* (XP_027078282.1); (**B**) Phylogenetic analysis of Ca16R with its orthologues in other plant species including *Datura stramonium*; *Solanum dulcamara*; *Solanum tuberosum*; *Olea europaea*; *Solanum pennellii*; *Anisodus acutangulus*; *Nicotiana tabacum*; *Solanum lycopersicum*; *Petunia x hybrid*; *Lycium ferocissimum*; *Coffea arabica*.

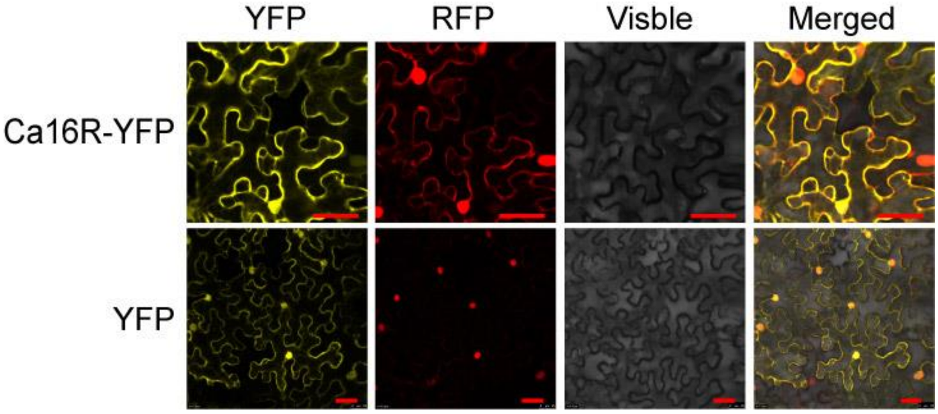

Figure S2. Subcellular localization of Ca16R in epidermal cells of *Nicotiana benthamiana* leaves. The *Nicotiana benthamiana* leaves were infiltrated with *A.tumefaciens* strain GV3101 containing the 35S:Ca16R-YFP construct. YFP and RFP fluorescence was imaged using a Confocal Microscope at 48 hpi. The histone NbH2B-RFP as a marker to indicate the nucleus and bar is 25 um.

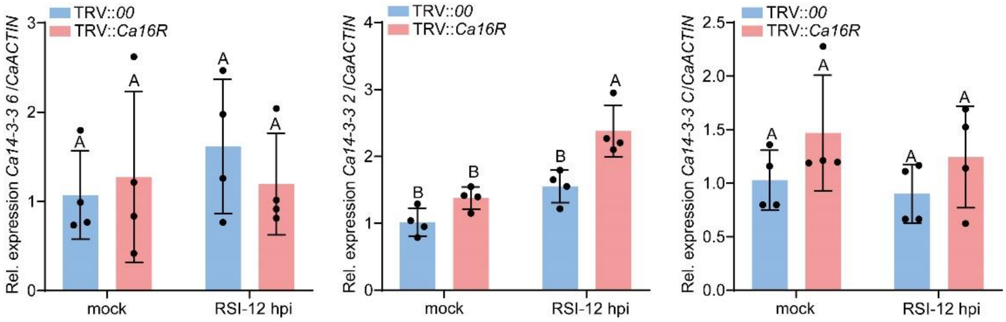

Figure S3. The specificity of Ca16R silencing. The RNA isolated from mock treated and RSI (at 12 hpi) challenged TRV:Ca16R and TRV:00 pepper plants were subjected to RT-qPCR using specific primer pairs of *Ca14-3-3 6*, *Ca14-3-3 2* and *Ca14-3-3 C*, which shares the highest sequence similarity to *Ca16R* among all members of 14-3-3 family in pepper, the effect of *Ca16R* silencing on transcript levels of *Ca14-3-3 6*, *Ca14-3-3 2* and *Ca14-3-3 C* were assayed. Data were shown as means ± standard error of four replicates. Different uppercase letters above the bars indicated significant differences between means ($P < 0.01$) by Fisher's protected LSD test.
